# “An epoch-making and blessed moment in the history of medicine” –thoughts on international health equity and the Nobel prize in medicine

**DOI:** 10.1186/s12939-021-01380-y

**Published:** 2021-02-10

**Authors:** Zachary M. Linneman, David J. Satin

**Affiliations:** 1 University of Minnesota Medical School, Minneapolis, USA; 2 Department of Family Medicine and Community Health, University of Minnesota, Minneapolis, USA

**Keywords:** Nobel prizes, Vaccine, COVID-19, Impact, Malaria, Public health, Global medicine, Research priorities, Health equity, Implementation science

## Abstract

The Nobel Prize in Physiology or Medicine is a prestigious award given every year for ostensibly the most important discovery in the field. Prizes in Medicine have typically gone to honor foundational knowledge rather than measurable impact. Two recent examples from global health (a rotavirus vaccine, child growth standards) offer alternatives for what might be lauded in medicine. These two examples and historical achievements regarding cholera and smallpox are worthy but do not fall within the scope of Nobel awards for Peace or Economics. The COVID-19 pandemic gives a new context for the idea that discovery and implementation are both keys to medicine. New patterns that redefine achievement in medicine could emerge by Nobel Prize precedent to promote greater health equity and international collaboration.

## History of the Nobel prize in medicine and other advances

In October, beginning with the category of Physiology or Medicine, the 2020 Nobel Prizes were announced. Rules for the Prize in Medicine permit it to be given to no more than three individuals (not an organization), all of whom must be living. This year awarded Alter, Houghton, and Rice for the discovery of the Hepatitis C virus [1]. Alfred Nobel’s last will and testament directs the Prize in Medicine to esteem “the most important discovery within the domain of physiology or medicine.” [2].

The late benefactor’s description presents as objective, but the Prizes in Medicine are miscible with history and politics. The second-ever Prize was awarded to Ross in 1902 for illuminating the malaria life cycle during a decade when malaria was commonly fatal on five continents, including to the British Army occupying India. In the lecture before the award to Erlich and Mechnikov in 1908 “for their work on immunity,” [3] Count K.A.H. Mörner invoked an earlier achievement in immunology 100 years before the Prize began, when Edward Jenner introduced cowpox vaccination for smallpox immunity. Mörner described that achievement as “an epoch-making and blessed moment in the history of medicine.” However, he followed his accolade by claiming that Jenner “did not advance the development of the study of immunity... the first and most important condition for making the problem of immunity the subject of real scientific research was namely to establish the cause of disease.” [4] In doing so, as Rector of the Royal Karolinska Institute (the awarding committee), he created an archetype for the Prize in Medicine that promotes the discovery of abstract knowledge - to the point of ideologically disqualifying the first-ever vaccine.

Following this pattern, Prizes in Medicine have typically gone to honor foundational knowledge (e.g. structure of the DNA molecule in 1962), innate bodily substance (insulin in 1923), or illumination of a disease-causing agent (HIV in 2008) [1]. Only one vaccine has ever been awarded [5]. The Prize consistently distinguishes moments in the history of medicine when knowledge first suggests the possibility of a cure or prevention of a disease, not the final realization of protection or cure in human bodies.

Alfred Nobel also offered general advice that all five original Prizes should be given “to those who, during the preceding year, have conferred the greatest benefit to humankind.” He would have stood in awe of crowning achievements of medicine and humanity after his lifetime: the discovery of sodium-glucose co-transport around 1960 leading to oral rehydration solution for cholera [6], the total eradication of smallpox in 1977 [7], and the 2006 development of a universal set of growth charts to assess malnutrition in children [8]. But the Nobel Prize in Physiology or Medicine did not award these undertakings.

These three accomplishments do not fit nicely in the established precedent or scope of the other Nobel Prizes. They are not directly related to war relief like the 1999 Nobel Peace Prize to Medicins San Frontieres (MSF), an organization which was founded in the aftermath of the Nigerian-Biafran War [9]. The three accomplishments are not innovations that directly impact the income potential of the poor, like Amartya Sen’s Economics award in 1998 “for his contributions to welfare economics”^1^ [11]. The closest precedent is this year’s Nobel Peace Prize to The World Food Programme (WFP) “for its efforts to combat hunger, for its contribution to bettering conditions for peace in conflict-affected areas … “ All of the non-medicine Nobel Prizes to health-related organizations are to non-governmental organizations (MSF and the Red Cross) or multilaterals (United Nations Children’s Fund, UNICEF, and WFP) and specifically laud accomplishments that are politically stabilizing in a period of war or potential conflict [1]. But where the three crowning achievements that we invoke diverge categorically (from non-Medicine Nobel Prizes) is that they are based on life-threatening diseases – cholera, smallpox, and severe acute malnutrition in children under five – all of which required medical intervention based on a new scientific discovery.

There is a crossing between discovery and impact forded by a tremendous amount of work. Ross described malaria in 1902 but treatment still failed to reach about 384,000 lives in 2019, two-thirds of whom are children [12]. Our historical high points begin in the mind, but do not self-assemble in the world. This is the work we will always have with us. One hundred and twenty years after a Nobel Prize for illuminating malaria’s life cycle, the pernicious agent of perennial health risk is confined to a subset of the world’s people. Many children in just a few parts of the world will typically suffer the course of this disease two or three times before developing natural immunity - if they survive.

## Recent Examples from Global Health of broad impact through medicine

Could we consider successful implementation of medical knowledge as its own discovery? Mörner’s discrimination towards *Eureka!* moments of abstract discovery in physiology is appealing precisely because it is akin to our conception of new love: immediately recognizable and often sentimentalized. But broad impact in medicine is more like the hard-earned intimacy of lifelong friendship, with origins often lost in private stories but fundamentally transformative over time, as described in the following two examples.

### An Indian-designed vaccine for a global virus

A vaccine for rotavirus (i.e. anti-diarrheal) was developed in India for infants [13]. Rotavac® began around 1988, when the late Dr. MK Bhan isolated strains of attenuated rotavirus from children at the All India Institute of Medical Sciences in New Delhi. The energetic Dr. Bhan, later as India’s Secretary of Biotechnology, built upon this discovery-turned-vaccine by facilitating a collaboration called the Biotechnology Industry Research Assistance Council (BIRAC), which brought the private research sector to Indian vaccine production.

Dr. Bhan’s primary *research* achievement is discrete, easy to explain, and traceable to a lightbulb moment, sitting in a hospital reviewing analysis of viral strains in diarrhea from infants. But the 30+ year *development* of the vaccine was a labor of love for many brilliant doctors, scientists, government officials, nurses, and field workers. Even more impactful is this vaccine’s *implementation*, whose rollout continues today with delivery and monitoring of Rotavac® in the Indian Public Health system [14]. Through medicine, children are spared a dangerous illness in large numbers.

### ‘WHO says our kids are just as good’

In 2004, Kofi Annan as Secretary-General of the United Nations wrote a preface to the World Health Organization (WHO) Multicentre Growth Reference Study (MGRS), noting “the United Nations undertook in 1993 a comprehensive review of anthropometric references” to “strengthen the hand of those working to extend the right to health to all children.” At least seven major research entities on five continents came together for a broad survey of those children having completely unrestricted physical growth (i.e. full food security, no major illness, optimal breastfeeding, non-smoking parents, etc) [8].

At the Asia site for this collaborative research project, Dr. Nita Bhandari led a team at what would become her remarkable female-led research organization called the Centre for Health Research and Development, Society for Applied Studies. They screened 117,000 pregnant women and followed up 425 of them at 73 different hospitals within 24 h of their child’s birth, diligently taking height and weight measurements of the children for years [15].

When results were combined from a representation of all geographic areas, a discovery of physiology was clear. Children have the same growth potential regardless of geography or the non-medical category of race. A newspaper headline in India summed up what applied to all areas where previous growth standards set the bar too low: “WHO says our kids are just as good.” [16] This discovery was implemented as the 2006 WHO Child Growth Standards, constituting an historic moment in medicine impossible to ascribe to three or fewer living people. The impact of the 2006 WHO Child Growth Standards was to substantially increase in the number of children accurately diagnosed with severe acute malnutrition (SAM). This in turn fostered a renewal in efforts to treat child malnutrition, including innovations in formulations for treatment and a new program funded by UNICEF called community-based therapeutic feeding for SAM [17, 18]. Through medicine, children are treated for a life-threatening illness in large numbers.

## A vision of the Nobel prize in medicine aligning with health equity

Since the Nobel Prizes began, medicine has evolved with a concept of health defined by the WHO ‘s founding documents in 1946 as “a state of complete physical, mental and social well-being and not merely the absence of disease or infirmity.” [19] Like a new growth standard that captures more cases of malnutrition, this definition of health comes with more work. But, global health inequities are more visible and collaborations to address them are more developed than when the Nobel Prizes began.

Can the Prize expand? The Nobel Prize in Medicine is without clear equal in the health sciences – it is a worthy aspiration for physicians and physiologists alike. And, like the highest award in any field, it helps define the culture and scope of the discipline. The Prize in Medicine also constitutes a record of historical achievements, and discoveries are usually awarded more than two decades after they are made (with a notable exception of insulin). [20] This is the long view of the Prize in Physiology or Medicine as an exercise in writing the history of medicine.

Successful physician-scientists plan their life work first by understanding history and hearing stories of achievements that came before them. Let them hear the long version – the version where impact is defined in counting the number of lives touched by a discovery implemented through collaboration and by prioritizing the disproportionately affected. It is the version in which doctors are driven to discover after being exposed to great need. Let them aspire to be humanitarians as well as discoverers. Through medicine, children in need can be spared or treated for serious illness on their way to a state of complete well-being – a broader definition of medicine that could be used by the Nobel Committee to expand the Prize’s territory, along with our maturing concepts of health and equity.

The reality of an uncontrolled pandemic due to a novel but well-characterized virus invites us to consider that discovering the cause of a disease may no longer be the most important concern for medical research. New patterns that redefine achievement in Physiology or Medicine could emerge. We write during a phase of the pandemic in which COVID-19 vaccines were rapidly developed and administered to a set of prioritized front-line healthcare workers in a limited number of wealthy nations. Yet the pandemic carries a lesson in health equity. “In an interconnected world, none of us is safe until all of us are safe,” UN Secretary-General António Guterres proclaimed in a speech early in the pandemic era [21]. The collaborations that produce and *broadly administer* an effective vaccine for COVID-19 would arguably meet both the original Nobel Prize criteria of “the most important discovery in the field” and having “conferred the greatest benefit to humankind. “And when the most severe restrictions of COVID-19 are lifted, our universal experience of uncertainty can unite us across physical distance and bring global health equity into public focus. Similarly, a Nobel Prize in Physiology or Medicine that honors a global health achievement from an area of greatest need could shift our values away from the ivory towers of research science towards impact.

Either that, or we can wait (like a quarter-million children), for a “blessed moment” when the eradication of malaria is achieved by combined efforts of novel vaccine development and a long vaccination campaign for an undeniably worthy *new* Nobel Prize Category. This prize could be endowed, perhaps, by a large and well-funded American pioneering organization in health science research with a fortune derived of a computer company - to honor “Global Medical Collaboration, Implementation, and Impact,” or simply, medical humanity.

## Data Availability

Not applicable.

## Abbreviations

BIRAC: Biotechnology Research Assistance Council
COVID-19: Coronavirus Disease 2019
MGRS: Multicentre Growth Reference Study
MSF: Medicins sans Frontieres (also as Doctors Without Borders)
SAM: Severe Acute Malnutrition
UNICEF: United Nations Children’s Fund
WFP: World Food Programme
WHO: World Health Organization

## Footnotes

1 The Economics prize, officially designated as the “Prize in Economic Sciences in Memory of Alfred Nobel,” was not delineated by Alfred Nobel’s will. It was added in 1968 with an endowment from the Swedish national bank [10].
